# Systematic Review of Parent-Youth Discrepancies in Exposures to Community Violence

**DOI:** 10.1007/s10567-025-00532-8

**Published:** 2025-08-11

**Authors:** Kajung Hong, Nicholas M. Morelli, Dalia R. Tabibian, Michelle G. Jimenez, David Straub, Miguel T. Villodas

**Affiliations:** 1 San Diego State University, University of California San Diego Joint Doctoral Program in Clinical Psychology, San Diego, CA USA; 2 Department of Psychology, San Diego State University, San Diego, CA USA; 3 Child and Adolescent Services Research Center, San Diego, CA USA

**Keywords:** Exposures to community violence, Informant discrepancies, Informant agreement, Victimization

## Abstract

Past studies have consistently found that different informants disagree on ratings of youth’s experiences. For instance, parents and youth report different prevalence and frequency ratings of youth’s exposure to community violence (ECV), with past studies demonstrating that parents typically underreport youth’s ECV compared to the youth. However, recent studies with advanced statistical analyses revealed more nuanced patterns of reports, with some parents overreporting their youth’s ECV, some underreporting it, and other parent-youth dyads agreeing that the youth either did or did not experience ECV. These report patterns are theorized to provide valuable insight into parent–child relationships and family functioning and have implications for youth emotional and behavioral development. The current systematic review synthesized 14 existing studies (*N* = 12,824 parent-youth dyads) on parent-youth discrepancies in youth ECV to elucidate patterns of informant discrepancies and their correlates to parent-youth relationship quality, family functioning, and youth outcomes. Studies that used advanced analytic approaches (k = 2), such as latent class analysis and polynomial regression, identified multiple patterns of parent-youth reports (e.g., parent-youth agreement on either low or high levels of youth ECV, parental underreporting, parental overreporting compared to youth). Poor parent-youth relationship and family functioning (e.g., lower parental warmth, higher parental hostility) were associated with higher parent-youth discrepancies in youth ECV. There were mixed findings with patterns of informant discrepancies in youth ECV and youth functioning. Suggestions for future directions for research on parent-youth discrepancies in youth ECV were made.

**Supplementary Information:**

The online version contains supplementary material available at 10.1007/s10567-025-00532-8.

## Systematic Review of Parent-Youth Discrepancies in Exposures to Community Violence

Exposure to community violence (ECV) has enduring, long-term effects on youth’s mental and physical health (Fowler et al., 2009; Wright et al., 2017). Despite the crucial public health importance of understanding ECV, discrepancies in reporting emerge, with parents and youth often providing different prevalence rates. In a 2010 synthesis on parent-youth discrepancies in reporting youth ECV, researchers found that parents typically reported lower levels of ECV than their youth (Goodman et al., 2010). Goodman and colleagues proposed the Discrepancies in Victimization Implicate Developmental Effects (DiVIDE) model to explain this pattern of discrepancies. They primarily attributed parent-youth discrepancies to limited disclosure by youth, which precludes parents from providing youth support and services, impacting youth coping and adjustment. Although this model provides an important foundation for understanding parent-youth discrepancies in youth ECV, it relies on the assumption that parent-youth discrepancies reflect parent underreporting and does not consider other patterns of parent-youth reporting (e.g., parent overreporting).

In the years since Goodman and colleagues (2010) proposed the DiVIDE model, advances in the application of quantitative research methods have facilitated studies of more nuanced patterns of parent-youth reporting discrepancies, as well as correlates of these discrepancies across a number of fields, including reports of youth psychopathology (e.g., Becker-Haimes et al., 2017) and family functioning (e.g., family cohesion or conflict; Xu et al., 2017). These methods provide an opportunity to extend the DiVIDE model and research on parent-youth discrepancies in youth ECV reporting (Laird & De Los Reyes, 2013; Laird & Weems, 2011). Additionally, recent studies have examined parent-youth ECV discrepancies using more diverse populations and advanced statistical methods, suggesting more nuanced patterns of parent-youth discrepancies than those synthesized by Goodman and colleagues previously. Therefore, the current systematic review synthesized the existing studies on parent-youth discrepancies in youth ECV to (1) identify additional patterns of parent-youth reports of youth ECV, (2) examine family and youth functioning as correlates of these patterns, and (3) make recommendations for future studies to extend the DiVIDE model.

## Exposures to Community Violence

According to Ecological Systems Theory (Bronfenbrenner, 1994), individual development is influenced by interactions and experiences in multiple proximal (e.g., families, peers, communities/neighborhoods, schools) and distal (e.g., society, media, culture) contexts. ECV encompasses exposure to direct victimization, witnessed violence, and vicarious exposure to intentional harm by strangers or acquaintances in the local neighborhood (Buka et al., 2001; Fowler et al., 2009). Violence includes acts of chasing, threatening, robbing, or causing physical harm/injuries (Brennan et al., 2007; Cooley-Quille et al., 1995; Guterman et al., 2000). Although there are similarities between violence exposure in community and school contexts, researchers have traditionally defined community violence as interpersonal violence in the immediate physical environment, excluding school violence exposure. This distinction arises from differences in contexts, perpetrators, methods of occurrence, and prevention methods compared to school violence. Community violence often involves criminal activities, such as assault, robbery, and gang violence, which are influenced by societal factors such as poverty, unemployment, substance abuse, and social unrest. It has a broader impact on the community and implications for policy and interventions within the neighborhood compared to school violence. Researchers have also traditionally distinguished ECV from exposure to violence in the media (e.g., witnessed violence on the internet, cyberbullying), which is more distal to the individual than directly experienced or witnessed violence.

Youth exposed to ECV face a heightened risk of developing long-term psychopathology and various physical health problems due to constant fear, psychological stress, and normalization of violence (Fowler et al., 2009; Wright et al., 2017). A review study found a significant association between ECV and PTSD among children and adolescents with a large effect (Fowler et al., 2009). Additionally, there was a significant association between ECV and externalizing symptoms with a moderate effect and a significant correlation between ECV and internalizing symptoms with a small-to-moderate effect (Fowler et al., 2009). Lastly, ECV had consistent, reliable associations with elevated blood pressure and sleeping problems (Wright et al., 2017).

## Importance of Examining Informant Discrepancies

Given the negative consequences of youth ECV, researchers have aimed to find accurate data on the prevalence of youth ECV. However, discrepancies frequently emerge between parent and youth reports of ECV, a phenomenon also similarly observed in assessments of other types of violence (e.g., child maltreatment; Cooley & Jackson, 2020), youth psychopathology or behavioral problems (e.g., De Los Reyes & Epkins, 2023), and parenting (e.g., Hou et al., 2020). These discrepancies were traditionally viewed as a threat to accurate data that undermined the credibility of scientific evidence. Conventional approaches to address informant discrepancies have included: (a) analysis that focused on shared variance with emphasis on the degree to which informants overlap in their ratings, (b) analysis that aggregated data from multiple informants into a single measured score, or (c) avoidance of multi-informants altogether and reliance on *optimal* informant, which can lead to under- or overestimation of the severity of the public health issues, misidentification of population needs, and wrong allocation of resources and funding (De Los Reyes et al., 2019).

However, informant discrepancies have been shown to reveal meaningful information. For instance, Cooley and Jackson (2022) conducted a systematic review of 13 studies that examined informant discrepancies in child maltreatment reports. Descriptive findings about higher youth-reported physical, sexual, and emotional abuse compared to caregiver-report or official case-report revealed important conclusions that contradicted the literature on the barriers to self-disclosure, such as shame (Cooley & Jackson, 2020). It highlighted the importance of sensitive screening of abuse experiences among youth to identify youth victims. In the case of youth behavioral problems, informant discrepancies have revealed variations in the expression of children's behaviors across different settings (e.g., school, home, lab; De Los Reyes, 2011). Similarly, researchers have proposed a theoretical model on parent-youth discrepancies in youth ECV and its link to maladaptive adjustment.

## The Discrepancies in Victimization Implicate Developmental Effects (DiVIDE) Model

In 2010, Goodman and colleagues synthesized past studies on parent-youth discrepancies in ECV. They examined nine studies on *parent-youth agreement* that used metrics of shared variance (e.g., kappa coefficients, Pearson *r* correlation), as well as three studies on *parent-youth discrepancies* that used difference score metrics. They concluded that most studies reported low shared variance between parents’ and youth’s reports of youth ECV, with parents typically reporting lower levels of youth’s ECV than youth themselves.

**Fig. 1:**
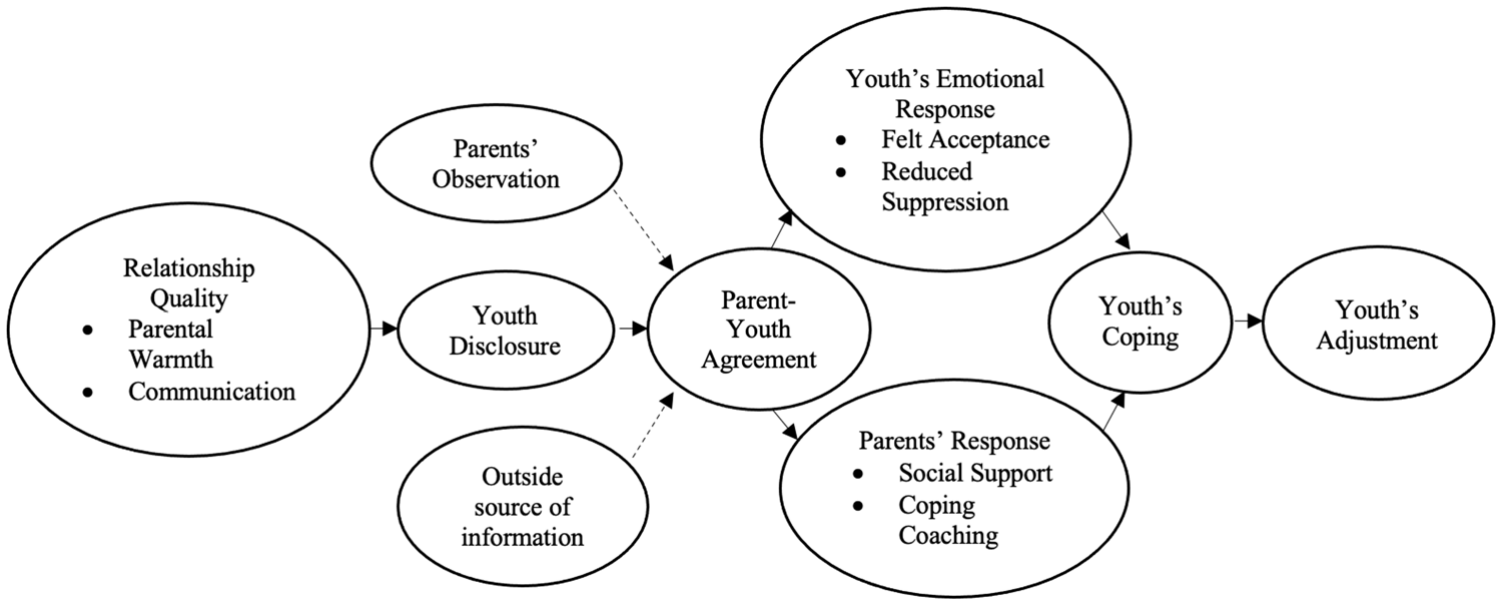
Summary of the DiVIDE model (Goodman et al., 2010)

Based on this previous research, Goodman and colleagues (2010) proposed the Discrepancies in Victimization Implicate Developmental Effects (DiVIDE) model (see Fig. 1) to elucidate the patterns of parental underestimation of youth’s ECV. As can be seen in Fig. 1, the DiVIDE model primarily attributes parent-youth discrepancies in reports of ECV to the lack of youth disclosure to parents. Though parents can gain information about their youth’s ECV through personal observation or external sources (e.g., police, teachers, or other parents’ reports), the model posits that parents predominantly rely on youth voluntarily sharing such information. In instances where parents have a negative relationship with their youth, characterized by a lack of closeness and communication, the youth might be reluctant to confide in their parents about ECV. According to the model, the lack of disclosure results in diminished parent-youth agreement on ECV, precluding parents from supporting their youth after ECV. This support could involve parents seeking services for youth or providing guidance about how to cope. Low agreement between parents and youth regarding ECV can lead to negative emotional responses in youth, such as low perceived acceptance by parents and increased emotion suppression. These emotional reactions may also contribute to maladaptive coping and maladjustment in youth. Taken together, the DiVIDE model linked low parent-youth agreement with poor family functioning and negative youth functioning.

## Limitations of the DiVIDE Model

Since Goodman et al. (2010) proposed the DiVIDE model, researchers have critiqued the use of difference scores for studying informant discrepancies (Laird & De Los Reyes, 2013; Laird & Weems, 2011). Although difference scores can be used descriptively to show relative discrepancy (Laird & Weems, 2011), researchers have argued studies examining difference scores only provide an overall impression of one pattern of discrepancies (e.g., parent underestimation of ECV) and limited information about the existence of multiple patterns of discrepant reports (e.g., parent-youth agreement about the existence of youth ECV, parent underestimation, parent overestimation). Further, relying on difference scores provides inconsistent and potentially misleading information when used in correlational or covariance-based analyses (Edwards, 2002; Laird & De Los Reyes, 2013; Laird & Weems, 2011). For instance, one cannot interpret whether parents’ reports, youth’s reports, or difference scores between parent-youth reports contribute to observed associations with correlates. Given that the DiVIDE model was based on studies that predominantly used measures of agreement and difference scores, it may need to be reexamined to adequately represent different patterns of parent-youth discrepancies in youth ECV.

Instead, researchers have suggested using polynomial regression with interaction terms, response surface analysis, or person-centered approaches (e.g., Latent Profile Analysis (LPA), Latent Class Analysis (LCA); Laird & De Los Reyes, 2013; Laird & Weems, 2011). These methods have been successfully applied to informant discrepancy studies in other research areas (e.g., parent-youth discrepancies in reports of family functioning, parenting, youth psychopathology; Laird & De Los Reyes, 2013; Shanock et al., 2010). For instance, in a meta-analysis and qualitative review of parent-adolescent informant discrepancy in parenting (Hou et al., 2020), 25 studies have utilized difference scores while more recent 11 studies have used polynomial regression with interaction terms. Furthermore, person-centered approaches may be particularly useful in identifying multiple nuanced patterns of parent-youth reports on youth ECV, as they identify distinct groups of individuals with similar patterns of traits (i.e., informant responses; Laursen & Hoff, 2006; Magnusson, 2003).

## Examining and Extending the DiVIDE Model

Recent studies on informant discrepancies in youth ECV considered important correlates across multiple ecological systems that were not previously considered in the DiVIDE model, such as neighborhood and cultural factors (e.g., neighborhood SES, race/ethnicity, immigrant generation status). Some studies also focused on subpopulations with collectivistic cultures that emphasize dedication, commitment, and loyalty to families, such as Chinese and Latino/a families (Alers-Rojas et al., 2020; Chan, 2015). Examining neighborhood and cultural influence may be important, as parent-youth discrepancies might relate to different constructs. For instance, families from collectivistic cultures might have higher expectations for youth disclosures, which might lead to higher parent-youth agreement. On the other hand, youth in collectivistic cultures may decide not to disclose ECV for fear of eliciting negative reactions from their parents and disrupting family harmony, resulting in higher parent-youth discrepancies on youth ECV reports. Additionally, cultural contexts might relate to other demographic correlates, such as gender and age, with different norms and expectations. For instance, parents of girls and younger youth may monitor their youth more closely in some cultures, leading to lower parent-youth discrepancies and lower ECV in general. Recent studies that considered ecological factors outside of the DiVIDE model can help us theorize additional factors and outcome variables related to parent-youth discrepancies in youth ECV as well as potential moderators in these associations for future studies.

## Current Study

It has been 14 years since Goodman and colleagues (2010) proposed the DiVIDE model. Since then, studies have examined correlates (e.g., family functioning, youth psychopathology) of parent-youth discrepancies in youth ECV with diverse populations and complex analyses, generating a new body of literature. However, no systematic review has synthesized and critically evaluated these studies. The current study aims to (1) identify multiple patterns of parent-youth reports of youth ECV, including discrepancies (e.g., parental over- and underestimation of youth ECV), (2) evaluate the existing DiVIDE model by examining family functioning and youth functioning as correlates, and (3) make recommendations for future studies to extend the DiVIDE model.

## Methods

### Selection of Manuscripts

The methodology for the current systematic review followed the latest PRISMA guidelines (Page et al., 2021). To obtain relevant manuscripts, electronic database searches, using PsychInfo and PubMed were conducted. For each database, the following search terms were used: (discrepan* OR disagreement OR discordance OR divergence OR incongruence OR underreport OR underestimation OR overreport OR overestimation) AND (parent* and youth* OR parent* and adolescent* OR parent* and child* OR parent-youth OR parent-adolescent OR parent–child OR informant*) AND (youth victimization OR community violence OR exposure* to violence OR violence exposure*). The same searches were also run in each database replacing parent with caregiver, mother, maternal, father, and paternal. The keywords were generated from past studies and suggestions from researchers with expertise in the field.

Additionally, we also reviewed all studies that cited Goodman et al. (2010). A snowball technique following guidelines from Wohlin (2014) was utilized. Reference lists of included studies were screened *backward* (i.e., references sections were studied to identify more studies) and *forward* (i.e., studies citing the selected studies were evaluated). Gray literature, such as dissertations and theses, was included.

## Inclusion and Exclusion Criteria

Articles were included if the following inclusion criteria were met: (a) the sample included parent-youth dyads, (b) parent-youth discrepancies in youth ECV were found, encompassing youth’s victimization, witnessed violence, and hearing about/vicarious exposure to violence in their neighborhood/community (Buka et al., 2001), (c) the article described an empirical study, (d) the article was published in a peer-reviewed journal or as a thesis or dissertation, and (e) the article was available in English. To allow for the broadest possible selection of articles, we did not limit the age of youth or the population (community vs. at-risk samples, such as youth in a residential treatment center). Moreover, we did not limit the methodology by which ECV was assessed, which ranged from self-report questionnaires to small group interviews. Exclusion criteria included: (a) the sample relied on either parents’ or youth’s reports, (b) the study examined informant reports on constructs that are not ECV, as defined in previous studies (e.g., parenting, perception of violence, domestic violence, school bullying, cyberbullying; Buka et al., 2001; Fowler et al., 2009), (c) the article only reported parent-youth agreement on youth ECV using shared variance metrics (e.g., Pearson *r* correlation*,* kappa coefficients), and (d) the article was nonempirical, such as narrative or meta-analytic reviews, theoretical papers, or technical papers. Articles that only reported parent-youth agreement on youth ECV using shared variance metrics were excluded because (1) these metrics provide limited and often misleading information on parent-youth agreement on youth ECV (i.e., a high agreement might reflect consistent disagreements between informants) and (2) a total of 12 articles were identified and 9 of them were already synthesized in Goodman et al., 2010. The overall finding of low levels of shared variance between parent-youth reports of youth ECV was unchanged.

## Selection of Studies

**Fig. 2:**
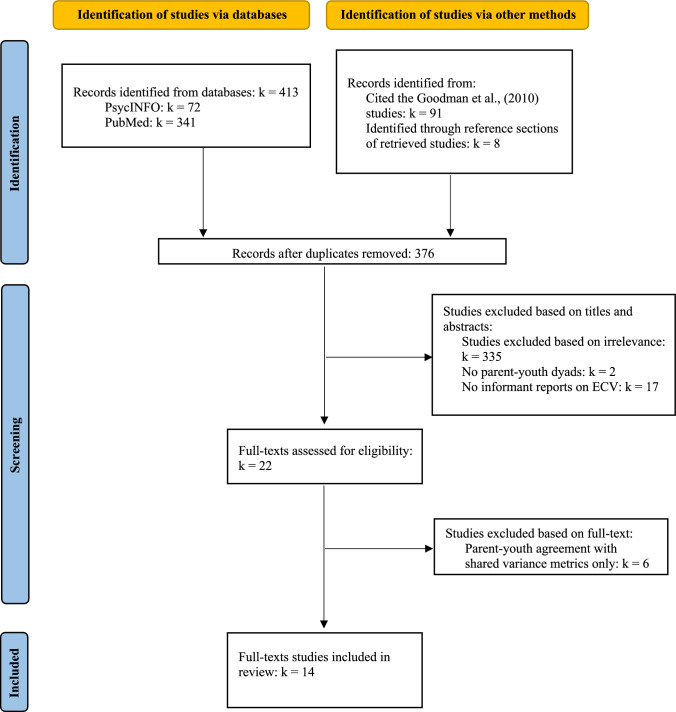
Flow chart of study selection process

The articles were screened by the first author. After the initial screening of the titles and abstracts, the first author retrieved full texts of articles to fully determine whether the articles met the inclusion criteria. The last author then reviewed and confirmed the inclusion of all articles retained based on their abstracts and titles. Disagreements about the inclusion of articles were handled through discussion between the first and the last authors. Endnote was used as the bibliographic software. Figure 2 presents the process of study selection.

## Quality Assessment

Study quality was assessed using modified checklists from the Joanna Briggs Institute (JBI; Moola et al., 2020). Cross-sectional studies were evaluated using a 7-item modified JBI Checklist for Analytical Cross-Sectional Studies and longitudinal studies were evaluated using an 11-item modified JBI Checklist for Cohort Studies (Moola et al., 2020). Both modified checklists included items about clearly defined inclusion criteria, as well as study subjects and settings, the use of valid and reliable measurements, the utilization of appropriate statistical analysis to examine informant discrepancies (e.g., polynomial regression with interaction terms, LCA/LPA), and the identification/controlling for confounds in correlational analyses. Other items that were irrelevant to the current literature (e.g., comparing two groups and examining whether the groups were similar and recruited from the same population) were omitted. The first author and two co-authors assessed the quality of each study reaching the final interrater reliability of ICC = 0.95. The modified JBI checklists are included in supplementary Tables S1 and S2. The percentage scores for acceptability of study quality and violations are reported in the last column of Table 1.

**Table 1:** Summary of studies examining parent-youth discrepancies

Study		Sample characteristics		Sample size (*N*), mean ages (*M*), age ranges (*R*)	Sex/gender (male %)		Measures	Procedure	Analyses on parent-youth discrepancies	Key findings with patterns of discrepancies	Correlates of discrepancies	Quality of studies acceptability (%), violation items
Studies that used recommended analysis for discrepancy reports (e.g., polynomial regression with interaction terms, response surface analysis, LCA/LPA)												
Goodman (2013)		Parent-youth in the Project on Human Development in Chicago Neighborhoods (PHDCN) comprised of diverse populations (33.2% Hispanics and 37.4% Black)		*N *= 485 Youth: *M *= 12.83 (*SD* = 1.60); *R *= 10-15	58.1% male youths No report of parents’ gender		6 victimization items in My ETV (Selner-O’Hagan et al. 1998) to assess prevalence of ECV in the last year	One-on-one interviews	Latent Class Analysis	3 classes with (1) “relative agreement” (54.5%) (2) “parents’ overestimation” (24%) (3) “parents’ underestimation” (21.5%) Excluded cases where dyads agreed on absence of victimization	Female youth more likely to be in discrepant classes. Younger youth more likely to be in discrepant classes. Racial differences in classes noted. *N.s* neighborhood SES differences in classes Mixed findings with youth psychopathology depending on informant for youth psychopathology.	Acceptability: 91% Longitudinal study checklist violation: Item 2 unclear (parents’ gender and age not reported)
Turnidge-Halvorson (2018)		Female caregiver-youth dyads identified as at-risk for child maltreatment from LONGSCAN, with diverse population (55% Black, 6% Hispanic)		*N* = 854 Youth age 8, 12 timepoints	47.5% male youth at age 8 Female caregivers (primarily biological mothers; 94.5%)		History of Witnessed Violence (LONGSCAN, 1988), Child’s Life Events Scale (LONGSCAN, 1992) at youth age 8 to measure summed total of prevalence of ECV in last year	One-on-one interviews	T tests Polynomial regression, response surface analyses	Female caregivers’ underestimation witnessed violence incidents (*t*(574) = 21.13, *p *< .001) Approx. a third of caregivers’ overestimation, a third of caregivers’ underestimation, a third of similar levels of report	Youth gender (boys) and history of caregiver victimization predictor of discrepancies Overall findings: parent-youth agreement on high exposure was associated with highest concurrent and future (4 years) psychopathology. Parent- youth agreement on low exposure was associated with lowest concurrent and future (4 years) psychopathology	Acceptability: 91% Longitudinal study checklist violation: Item 2 unclear (caregiver age not reported)
Studies that used other analysis for discrepancy reports (e.g., difference scores, chi-squared difference, McNemar’s test)												
Alers-Rojas et al. (2020)		Latino mothers-adolescents in urban neighborhoods from the Project on Human Development in Chicago Neighborhoods (PHDCN)		*N *= 287 Youth: T1 (age 11): *M *= 11.22 (SD = .59); T2 (age 14) *M*= 13.69 (SD = .55) Mothers T1: *M *= 35.30 (SD = 6.09)	53% male youth All mothers		8 witnessed violence and 6 victimization items in My exposure to violence (My ETV; Selner-O’Hagan et al. 1998) to assess prevalence of ECV in the last year	One-on-one or phone interviews at two time points	Summed agreement scores (0 = disagree, 1 = agree)	Witnessed T1: *M* = 13.33 (SD = 2.74), [3-16] Witnessed T2: *M* = 13.26 (SD = 2.28), [3-16] Victimization T1: *M* = 11.55 (SD = 1.15), [4, 12] Victimization T2: *M* = 11.50 (SD = 1.11), [4, 12] Mothers’ overestimation at age 11 Mothers’ underestimation at age 14	General findings: higher agreement significantly correlated with lower concurrent and future (3 years) youth-reported internalizing and externalizing symptoms.	Acceptability: 91% Longitudinal study checklist violations: Item 11 (recommended analysis for discrepancy reports include LCA/LPA, polynomial regression)
Ceballo et al. (2001)		Female caregiver-youth with diverse population (53% Hispanic, 13% Black) from predominantly low SES backgrounds (71% welfare history)		*N* = 104 Youth: *M *= 10 *R* = Grades 4–5 Caregivers: *M *= 36	46% male youths All female caregivers		Witnessed violence and victimization items on Survey of children’s exposure to violence (SCEV; Richters & Martinez, 1990) to assess lifetime prevalence of ECV	Small group interviews for youth, One-on-one interviews for female caregivers	Chi-squared differences in prevalence rate	Witnessed violence: almost all items significant ranging from 5.35 (*p *< .05; witnessed gun violence) to 27.16 (*p *< .001; witnessed suicide) Victimization: all items significant ranging 4.24 (*p *< .05; burglary) to 15.32 (*p *< .001; threatened) Overall caregiver underestimation	Higher agreement for younger, female youth compared to older, male youth. Other demographics (e.g., marital status, employment) *ns.* Higher agreement significantly associated with fewer PTSD and internalizing symptoms.	Acceptability: 91% Cross-sectional study checklist violations: Item 11 (recommended analysis for discrepancy reports include LCA/LPA, polynomial regression)
Chan (2015)		Parent-adolescent dyads in six cities in China		*N *= 2624 Youth: *M *= 16.1 (SD = .8) R = 15 -17 Parents: Primarily age 35–54	52% male youths No report of parents’ gender		Witnessing or indirect victimization (excluding domestic violence) prevalence subscales from the Chinese version of the Juvenile Victimization Questionnaire (JVQ)	Questionnaires	Chi-squared differences, Percentage of agreement, disagreement (dichotomous scores)	χ^2^ (1, *N *= 2624) ranges from 37.2 (stolen at home) to 170.4 (witnessed murder) Percentage of agreement: 88.4% - 98.4% Percentage of disagreement: 1.6% - 16.7% Overall parental underestimation	N/A	Acceptability: 72% Cross-sectional study checklist violation: Item 2 unclear (parents’ gender not reported) Item 11 (recommended analysis for discrepancy reports include LCA/LPA, polynomial regression)
Hill and Jones (1997)		Parent-youth dyads in high (*n* = 51) and low-violence (*n* = 45) communities in Washington, DC, U.S (racial breakdown not reported)		*N *= 96 Youth: *R *= 9–13	52% boys		Parent and youth interviews (different questions) on witnessed community violence in past 3 years	One-on-one interviews	Chi-squared differences	Witnessed homicides: *χ*^2^ (1, *N *= 96) = 6.03, *p *< .05 Witnessed non-fatal shooting: *χ*^2^ (1, *N *= 96) = 5.36, *p *< .05 Predominantly parents’ underestimation	More agreement on more deadly crimes Youth with more discrepancies had parents perceived less peer support No other demographics differences in discrepant vs. agreement group (e.g., income, maternal edu., child anxiety)	Acceptability: 29% Cross-sectional study checklist violations: Item 2 unclear (parents’ gender, age, youth race not reported) Item 3 (psychometric for interviews on witnessed community violence not sufficiently tested in past studies) Item 4,5 (no other covariates considered in link between discrepancies and peer support) Item 11 (recommended analysis for discrepancy reports include LCA/LPA, polynomial regression)
Howard et al. (1999)		Caregiver-youth dyads in low-income public housing developments in the East Coast., U.S. predominantly African Americans (96%) and mostly without two biological parents (71%)		*N* = 349 Youth: *R* = 9–15 Parents: *M *= 36 (reported significant missing data with parental age)	57% male youths 84% female parents		17 witnessed community violence and 12 victimization items on modified SCEV (Richters & Saltzman 1990) to measure frequency of ECV in the past 6 months	Q uestionnaires	Mean difference scores Absolute agreement scores resulting in 3 separate groups with (1) “low (<50%) agreement,” (2) “moderate (50-80%) agreement,” (3) “high (>80%) agreement”	Witnessed violence: Overall caregiver overestimation (24%) and caregivers’ underestimation (53%) Victimization: Overall caregivers’ underestimation (58%) (1) “Low agreement” (26%) (2) “moderate agreement” (56%) (3) “high agreement” (18%)	Significant differences in youth family functioning, psychosocial pathology (violence perpetration, risks), social competence with general trend of worse outcome for “low agreement groups” compared to others	Acceptability: 86% Cross-sectional study checklist violation: Item 11 unclear (recommended analysis for discrepancy reports include LCA/LPA, polynomial regression)
Lewis et al. (2012, 2013)		Caregiver-youth dyads identified as at-risk for child maltreatment from Longitudinal Studies of Child Abuse and Neglect (LONGSCAN), with diverse population (55% Black, 6% Hispanic)		*N = *766 Youth: *M* = 12.4 (SD = 0.44) *R* = 11–13.8	50% male youths 94% female caregivers		History of Witnessed Violence measure (LONGSCAN, 1998) and modified Child’s Life Events Scale (LONGSCAN, 1992) to match History of Witnessed Violence. Questionnaires measure prevalence of ECV in the past year	One-on-one interviews	McNemar’s difference test (1) “neither report” (2) “both report” (3) “youth only report” (4) “parent only report	χ² (1, *N* = 766) = 129.1, *p* < .05 (1) 51% “neither report” (2) 8% “both report” (3) 34% “youth only report” (4) 7% “parent only report”	Demographics (e.g., youth gender, race, income) not associated with parent-youth agreement. “Both report” and “youth only report” groups higher on youth-reported trauma symptoms and youth delinquency than “parent only report” “Both report” group higher on counseling service needs than other groups, however *ns* for the use of counseling services “Youth only report” group reported less youth-reported parental monitoring than “parent only report” group	Acceptability: 57% Cross-sectional study checklist violation: Item 2 unclear (parents’ age not reported) Item 6 unclear measures of service needs and utilization developed by LONGSAN researchers without adapting them from previous findings; however, these are one-item measures with high face validity Item 11 unclear (recommended analysis for discrepancy reports include LCA/LPA, polynomial regression)
Raviv et al. (2001)		Mother-youth dyads with 2^nd^ and 4^th^ graders in low (*n *= 65) and high violence (*n *= 69) neighborhoods in Tel Aviv, Israel		*N *= 134 Youth: *R *= 2nd and 4th graders	50% boys All mothers		VEX-R (Fox & Leavitt, 1995) to measure frequency of ECV in lifetime	Questionnaires	Mean difference (Mother reports – youth reports)	Mean difference scores (mother reports – youth reports) = 019 Mothers’ overestimation of youth mild violence exposure in neighborhood	N/A	Acceptability: 71% Cross-sectional study checklist violation: Item 2 unclear (parents’ age not reported) Item 11 (recommended analysis for discrepancy reports include LCA/LPA, polynomial regression)
Richters and Martinez (1993)		Caregivers-youth dyads in low-income, moderately violent urban neighborhood in Southeast Washington D.C., U.S. (97% Black)		*N* = 165 Youth: *R* = 6–10 Grades 1–2: *n* = 111 Grades 5–6: *n* = 54	51% male youths 79% mothers 9% fathers 11% other relatives		SCEV (Richters & Saltzman, 1990) for parents and 5 – 6^th^ graders Things I have seen and heard (Richters & Martinez, 1990) for 1–2nd grade students to measure frequency in the past 5 years (or lifetime for younger youth)	Small group interviews for youth Questionnaires or one-on-one interviews for parents depending on reading levels	Chi-squared difference of prevalence rates	Older youth lifetime victimization: 69% youth reports vs. 44% parent report, χ² (1, *N* = 54) = 4.59, *p *< .05 Younger youth witnessed violence: χ² (1, *N* = 111) ranging from 17.40 *(p < *.001; witnessed arrests) – 58.48 *(p < *.001; witnessed serious accidents) with parental underestimation; Witnessed stabbing and drug deals *n.s.*	N/A	Acceptability: 71% Cross-sectional study checklist violation: Item 2 unclear (parents’ age not reported) Item 11 (recommended analysis for discrepancy reports include LCA/LPA, polynomial regression)
Skar et al. (2021)		Caregiver-youth dyads assessed in Norwegian child and adolescent mental health services between 2012 and 2017		*N *= 6094 Youth: *M* = 12.03 (SD = 3.14) *R = *6–18	47% boys		Community violence items from trauma screening checklist using DSM-5 (Jensen et al., 2014)	Clinical screenings	Chi-squared difference McNemar test (1) “neither report” (2) “both report” (3) “caregiver only report” (4) “youth only report	Statistics of chi-squared difference test not specified except caregiver report: 35.2%, youth report: 42.6%, *p *< .001 (1) 46.9% “neither report” (2) 24.7% “both report” (3) 10.5% “caregiver only report” (4) 17.9% “youth only report”	*“*Both reporting” associated with higher post-traumatic stress symptoms and functional impairment compared to “caregiver only report” or “youth only report”	Acceptability: 57% Cross-sectional study checklist violations: Item 2 unclear (parents’ age and gender not reported) Item 5 covariates (youth age, gender not controlled) Item 11 unclear (recommended analysis for discrepancy reports include LCA/LPA, polynomial regression)
Strudler (2003)		Caregiver-youth dyads in residential treatment center in urban Massachusetts, U.S. (18.3% Black, 15% Hispanic)		*N *= 60 Youth: *R *= 6–17	51.7% male youths		Things I have seen and heard (Richters & Martinez, 1990), SCEV (Richters & Saltzman, 1990) to measure frequency	One-on-one interviews	Dichotomous scores (1) “Agreement” (2) “Parents’ overestimation” (3) “Parents’ underestimation”	(1) “Agreement” (55%) (2) “Parents’ overestimation” (13%) (3) “Parents’ underestimation” (25%) χ² (2, *N* = 56) = 17.82, *p* < 0.001	N/A	Acceptability: 71% Cross-sectional study checklist violation: Item 2 unclear (caregiver age and gender not reported) Item 11 unclear (recommended analysis for discrepancy reports include LCA/LPA, polynomial regression)
Zimmerman (2014)		Parent-youth dyads in urban neighborhoods from PHDCN study in age 9, 12, 15 cohorts with diverse population 35.8% Black, 45.8% Hispanic)		*N = *2344 Youth: *M *= 12.0 at baseline (SD = 2.4) *R = *7.8–16.9	50.4% male youths 83% biological mothers, 9% biological fathers		9 secondary ECV items in SCEV (Richters & Saltzman, 1990) to measure last year prevalence at T2 interviews	One-on-one interviews	Absolute and directional mean difference scores (youth reports – parent reports)	Absolute mean difference scores (youth reports – parent reports): *M *= 1.7 (SD = 1.6) [0, 9] Directional mean difference scores (youth reports – parent reports): *M *= 1.0 (SD = 2.1) [− 7, 9]	More discrepancies associated with demographic factors (e.g., males, non-whites, older youth, 3rd generation immigrants, without two biological parents, without married parents, lower SES) More discrepancies associated with lower family functioning and higher risks at T1 interviews.	Acceptability: 81% Longitudinal study checklist violation: Item 2 unclear (parents’ age not reported) Item 11 (recommended analysis for discrepancy reports include LCA/LPA, polynomial regression)
Zimmerman and Farrell (2013), Zimmerman and Pogarsky (2011)		Parent-youth dyads in urban neighborhoods from PHDCN study in age 12 and 15 cohorts with diverse population 33% Black, 51% Hispanic		*N = *1517 Youth: *M *= 13.5 baseline	49.11% male youths 82% biological mothers, 10% biological fathers		10 items on modified SCEV (Richters & Saltzman, 1990) to measure lifetime prevalence of witnessed violence	One-on-one interviews	Overall difference scores (parent-youth), % in (1) “parent-youth agreement” (2) “parents’ overestimation” (combined high and low parents’ overestimation) (3) “parents’ underestimation” (combined high and low parents’ underestimation)	(Parent – male youth) difference scores: − 1.9 (Parent – female youth) difference scores: − 1.6 Overall: (1) 12% “parent-youth agreement” (2) 22% “parents’ overestimation” (3) 66% “parents’ underestimation” Male youth: (1) 11.5% “parent-youth agreement” (2) 19.5% “parents’ overestimation” (3) 68.5% “parents’ underestimation” Female youth: (1) 13.2% “parent-youth agreement” (2) 24% “parents’ overestimation” (3) 62.8% “parents’ underestimation”	Gender differences in parent–youth discrepancies were modest and did not reach significance “High parents’ underestimation” group associated with future (3 years) internalizing, externalizing general offending Weak partial mediation between parents’ underestimation and psychopathology by family support	Acceptability: 81% Longitudinal study checklist violation: Item 2 unclear (parents’ age and gender not reported) Item 11 unclear (recommended analysis for discrepancy reports include LCA/LPA, polynomial regression)

## Results

### Study Characteristics

A total of 16 articles met the inclusion criteria for the current review (see Table 1 for details about articles). However, some articles, such as Zimmerman and Farrell (2013) and Zimmerman and Pogarsky (2011) as well as Lewis et al. (2012, 2013) used the same sample, timepoints, and ECV measures; these *articles* were considered to be one *study* (k) for this review. Throughout, we report the number of studies (k), out of 14, that used non-identical samples.

The study selection procedure is illustrated in Fig. 2. The 14 studies were drawn from 10 unique datasets. Four studies (Alers-Rojas et al., 2020; Goodman, 2013; Zimmerman, 2014; Zimmerman & Farrell, 2013; Zimmerman & Pogarsky, 2011) utilized longitudinal data from the Project on Human Development in Chicago Neighborhoods (PHDCN; Earls & Visher, 1997; Earls et al., 2007). Almost all of these studies used data from either waves 1 and 2 or waves 2 and 3. Given the overlap in samples and timepoints, findings from these PHDCN studies should be interpreted with caution. Two studies (Lewis et al., 2012, 2013; Turnidge-Halvorson, 2018) utilized data from the Longitudinal Studies of Child Abuse and Neglect (LONGSCAN; Runyan et al., 1998). The remaining 8 studies used independent datasets.

Seven studies reported both victimization and witnessed violence, whereas six studies reported witnessed violence only (Chan, 2015; Hill & Jones, 1997; Lewis et al., 2012, 2013; Turnidge-Halvorson, 2018; Zimmerman, 2014; Zimmerman & Farrell, 2013; Zimmerman & Pogarsky, 2011) and one study reported victimization only (Goodman et al., 2010). Additionally, 9 studies used concurrent study designs or a single timepoint from a longitudinal study; five studies using either PHDCN or LONGSCAN datasets utilized longitudinal analyses (Alers-Rojas et al., 2020; Goodman, 2013; Turnidge-Halvorson, 2018; Zimmerman, 2014; Zimmerman & Farrell, 2013; Zimmerman & Pogarsky, 2011).

## Quality Assessment

The quality of studies included in this review is presented in Table 1. None of the studies achieved 100% acceptability scores as most studies used analytic strategies not recommended by the literature (examples of recommended analytic strategies include polynomial regression and LCA / LPA; Laird & De Los Reyes, 2013; Shanock et al., 2010) and did not report parental demographic information (e.g., gender, age, race). As can be seen in Table 1, seven studies scored above 80% and four more studies scored around 70%. One article had a 29% acceptability score because it failed to report parental demographic information, used a non-validated youth ECV assessment, used methods of analyzing discrepancies that are not recommended, and did not identify or control for confounds when assessing correlates of parent-youth discrepancies in youth ECV (Hill & Jones, 1997). Studies with lower quality should be interpreted with caution.

## Sample Characteristics

The current systematic review included a total of 12,824 unique parent-youth dyads. Sample sizes ranged from 60 to 6094. As reported in Table 1, ten studies included predominantly Black and Hispanic parent-youth dyads living in urban U.S. neighborhoods. Three studies included parent-youth dyads in China, Israel, and Norway. Youth were well-balanced regarding sex/gender (i.e., male youth ranging from 46 to 58.1% of *N*). Youth ages ranged from 6 to 18 but were primarily focused on middle childhood or adolescence. Parental demographics (e.g., age, race/ethnicity) were not sufficiently reported in most studies (*k* = 11). However, most studies consisted of predominantly female caregivers (i.e., over 80%). Among the three studies that reported parents’ age, two reported means of approximately 35 years and one reported that “most” parents were within 35–54 years of age. Most participants were recruited from communities or schools (*k* = 12). The remaining samples consisted of outpatients from a mental health clinic or inpatients at a residential treatment center. Additionally, youth from LONGSCAN (*k* = 2) were identified as at-risk for child maltreatment.

## Assessment of Youth ECV

A variety of measures were used to assess youth ECV, typically via one-on-one interviews or written questionnaires. Table 1 shows that in 9 studies, parents and youth completed the same youth ECV measures, whereas in five studies, parents and youth were administered different measures of youth ECV with similar items. Many studies reported the prevalence of ECV (*k* = 9) while fewer studies (*k* = 5) reported the frequency of community violence exposures.

## Analytic Approach

Table 1 demonstrates that multiple analytic approaches were used to examine parent-youth discrepancies in youth ECV and their correlates, ranging from mean difference scores to more complex analyses, such as LCA and regression analysis with interaction terms (e.g., Laird & De Los Reyes, 2013). Mean difference scores were used in four studies, while chi-squared difference tests using prevalence scores were used in five studies. Two studies used approaches involving agreement scores (i.e., 0 = discrepant, 1 = agreement; mean agreement scores summed and represented as percentages of agreement/disagreement). Five studies reported more detailed findings involving percentages of different parent-youth response patterns (e.g., “both parent-youth agree on youth’s ECV,” “only parents report ECV,” “only youth report ECV”) that resembled or used McNemar’s test. Some of these studies differentiated parents and youth agreeing on high/presence of ECV and low/absence of ECV (*k* = 2), whereas others did not differentiate and just reported on agreement (*k* = 3). Lastly, one study used LCA to examine different patterns of parent-youth responses on youth ECV (Goodman, 2013), while another used polynomial regression analyses with interaction terms as well as response surface analysis (Turnidge-Halvorson, 2018).

## Key Findings

### Patterns of Parent-Youth Discrepancies in Youth Exposure to Community Violence

Studies that used mean difference scores and chi-squared tests typically found that parents significantly underestimated ECV compared to their youths’ reports. One partial exception was Raviv et al. (2001), which identified the typical pattern of parental underestimation in the full sample, but found significant *overestimation* among mothers of younger children (8–10 years) regarding exposure to mild violence in the neighborhood.

Analyses identifying more nuanced patterns of reports (e.g., McNemar’s test) often found that the majority (i.e., above 50%) of parent-youth dyads reported similar prevalence or frequency of youth ECV. The exceptions were studies that used the PHDCN dataset, which found low agreement (12% of parent-youth dyads) and the majority of parents reporting higher rates of lifetime prevalence of witnessed violence than their early adolescents (66% of parent-youth dyads; Zimmerman & Farrell, 2013; Zimmerman & Pogarsky, 2011). Parents and youth were more likely to agree on a lack of ECV (Lewis et al., 2012, 2013; Skar et al., 2021). This pattern of agreement on non-occurrence is expected for events with less frequent base rates. However, other studies did not differentiate whether parent-youth agreement was regarding high levels, low levels, or absence of youth ECV (Howard et al., 1999; Strudler, 2003; Zimmerman & Farrell, 2013; Zimmerman & Pogarsky, 2011). Between parental underestimation and overestimation, the former pattern was more prevalent (ranging from 17.9 to 66%) compared to the latter (ranging from 7 to 24%). With one exception (Strudler, 2003), studies often reported the discrepancy patterns descriptively with percentages but did not conduct tests of statistical significance to see whether the parents’ and youth’s report patterns significantly differed.

Goodman (2013) conducted LCAs using the PHDCN sample of 9 and 12-year-old children and their parents who reported past-year prevalence of youth victimization. Three classes were identified based on patterns of parent-youth agreement: “relative agreement” (54%), “parental overestimation” (24%), and “parental underestimation” (21.5%). The authors did not test whether the three classes differed significantly from one another in the degree of their discrepancy. Dyads in the “relative agreement” class agreed on youth experiencing one or more instances of victimization in the past year, though youth still tended to report more victimization than parents. For both “parental overestimation” and “parental underestimation” classes, the indicator “youth being hit” was a major differentiator. In the “parental overestimation” class, the probability of parents reporting that their youth was hit was 100% compared to 17% for youth, whereas in the “parental underestimation” class, the probability of parents reporting that their youth was hit was 4% compared to 100% for youth.

Finally, Turnidge-Halvorson (2018) used polynomial regression with interaction terms to test the correlates of parent-youth discrepancies in youth witnessing violence in the past year among caregiver-youth dyads from the LONGSCAN sample when youth were 8 years old. T-tests demonstrated a general pattern of parental underestimation of youth who witnessed violence. The author also described a full range of parent-youth discrepancy patterns with one-third of the sample demonstrating parental underestimation, one-third demonstrating parental overestimation, and the rest demonstrating general agreement; however, they did not report any statistical tests that supported this statement. Results from the polynomial regression are described in the subsequent section.

### Correlates of Parent-Youth Discrepancies in Youth Exposure to Community Violence

**Demographics and Family Background**. Demographic correlates of parent-youth discrepancy patterns, such as youth age, gender, race/ethnicity, immigrant generation, socioeconomic status (SES), caregiver education, and caregiver victimization history, were reported in six studies. According to two studies, older youth-parent dyads were more likely to report higher discrepancies than younger youth-parent dyads, measured via mean difference scores and absolute agreement scores (Alers-Rojas et al., 2020; Zimmerman, 2014). Findings related to gender were mixed. Two studies that used chi-squared difference tests and t-tests for parent-youth discrepancies generally found more parent underestimation in ECV reporting among male youth-parent dyads relative to female youth-parent dyads (Skar et al., 2021; Turnidge-Halvorson, 2018). On the other hand, two studies used mean difference scores (Zimmerman & Farrell, 2013) and McNemar’s test (Lewis et al., 2012, 2013) found no significant association between parent-youth discrepancy and gender. Lastly using LCA, Goodman (2013) found that female youth and their parents were more likely to be in discrepant classes (i.e., both parental overestimation and underestimation) compared to male youth and their parents. Thus, studies using different analytic approaches found varied results.

Findings were also mixed for race/ethnicity as a correlate. Using McNemar’s difference test, Lewis et al. (2012, 2013) reported no significant associations between race/ethnicity and parent-youth discrepancies in their sample of at-risk caregiver-youth dyads. On the other hand, Zimmerman (2014) reported that non-white, as well as third-generation immigrant youth and their parents, were more likely to demonstrate higher discrepancies based on mean difference scores. Using LCA, Goodman (2013) reported that Black youth and their parents were more likely to be in the parental overestimation class compared to the parental underestimation class, whereas Hispanic youth and their parents were more likely to be in the parental underestimation or agreement classes compared to the parental overestimation class.

Regarding SES and caregiver education, two studies found no significant association with discrete patterns of parent-youth ECV reporting discrepancy (Goodman, 2013; Lewis et al., 2012, 2013); however, one study that conducted a logistic hierarchical item response analysis found that parent-youth discrepancies using mean difference scores were a function of lower household income (Zimmerman, 2014). Lastly, one study identified an association between the severity of parent victimization history and the discrepancy of reports of youth witnessing violence, with parents with higher victimization history likely overreporting youth ECV (Turnidge-Halvorson, 2018).

Two studies reported on parent-youth discrepancies in ECV in demographic populations that traditionally endorse collectivistic cultural values (Alers-Rojas et al., 2020; Chan, 2015). Both reported dichotomous agreement scores (e.g., 0 = disagreement, 1 = agreement) and found high levels of agreement in youth who witnessed violence and victimization, as evidenced by a high percentage of agreement ranging from 88 to 98%. However, in a sample of Chinese parent-youth dyads, Chan (2015) found that parents reported significantly fewer instances of their youth witnessing violence compared to their youth’s reports based on chi-square tests. Alers-Rojas et al. (2020) descriptively identified a more complicated picture of parent-youth discrepancies among their sample of Latino-American mother-youth dyads, such that mothers overestimated their younger children’s ECV but underestimated their adolescents’ ECV; however, statistically significant differences were not tested. Neither study tested the association between discrepancies and collectivistic cultural values.

**Family Functioning**. Four studies examined the association between family functioning and parent-youth discrepancies in youth ECV (Howard et al., 1999; Lewis et al., 2013; Zimmerman, 2014; Zimmerman & Farrell, 2013; Zimmerman & Pogarsky, 2011). Overall, these studies found evidence that poorer family functioning (i.e., less support and open communication, less parental warmth) was associated with higher discrepancies in reports of youth ECV. In particular, the parental underestimation pattern identified using mean difference and McNemar’s tests was associated with lower parental warmth, support, and monitoring (Lewis et al., 2013; Zimmerman, 2014).

**Psychopathology and Functioning**. Nine studies reported associations between parent-youth discrepancies in youth ECV and youth functioning, including youth psychopathology. Studies that used mean difference scores, chi-squared difference tests, and total agreement scores found that lower parent-youth agreement and higher discrepancies were associated with worse parent- and youth-reported clinical outcomes (e.g., higher concurrent and future internalizing, externalizing, PTSD symptoms) as well as functioning (e.g., higher risk taking behaviors, lower peer support; Alers-Rojas et al., 2020; Ceballo et al., 2001; Hill & Jones, 1997; Howard et al., 1999). Findings from studies that reported more detailed and complex parent-youth response patterns (e.g., McNemar’s test, polynomial regression with interaction terms) were mixed, with two studies suggesting associations between parental underestimation and worse outcomes (e.g., internalizing, externalizing, trauma symptoms, delinquency; Lewis et al., 2012, 2013; Zimmerman & Farrell, 2013; Zimmerman & Pogarsky, 2011). One of these studies found that low family support mediated the association between parents’ underestimation of youth ECV and youth psychopathology (e.g., internalizing, externalizing problems, offending; Zimmerman & Pogarsky, 2011). However, two studies with higher risk samples found that parent-youth agreement on high levels of ECV is linked with worse outcomes (Skar et al., 2021; Turnidge-Halvorson, 2018). In a clinical setting, Skar et al. (2021) found that parent-youth agreement about the presence of ECV was associated with higher post-traumatic stress and impairment symptoms compared to parental overestimation or underestimation groups. Similarly, among families with a higher risk of child maltreatment, Turnidge-Halvorson (2018) found that parent-youth agreement on a high level of youth witnessing violence was associated with the highest concurrent and subsequent (4 years) psychopathology, whereas parent-youth agreement about low youth witnessing violence was associated with the lowest. Parent-youth agreement on youth ECV in this sample was associated with a greater identified need for counseling/clinical services but was not associated with the actual use of services (Lewis et al., 2012).

Lastly, a study that examined parent-youth discrepancies using LCA found mixed results depending on the source and developmental timing of youth outcomes (Goodman, 2013). The study found that dyads in the parental overestimation class had higher concurrent parent-reported aggression and higher subsequent youth-reported anxiety/depression (2.5 years) compared to dyads in parental underestimation or relative agreement classes. However, dyads in the parental underestimation and relative agreement classes had higher concurrent youth-reported anxiety/depression, aggression, and delinquency compared to the parental overestimation class.

## Discussion

The current systematic review synthesized existing studies on discrepant parent-youth reports of youth ECV to (1) identify additional patterns of parent-youth reports of youth ECV, (2) examine family and youth functioning as correlates of these patterns, and (3) make recommendations for future studies to extend the DiVIDE model (Goodman et al., 2010). The DiVIDE model was limited by relying on studies using analytic strategies for examining parent-youth discrepancies (e.g., difference scores) that were not capable of identifying more nuanced patterns of parent-youth discrepancies (e.g., parental over/underestimation, parent and youth agreement on absence/presence of ECV). We identified 14 studies published since the DiVIDE model was proposed examining parent-youth discrepancies in youth ECV. Though studies ranged widely in their assessment and analytic strategies, studies that applied advanced analytic strategies identified more nuanced patterns of parent-youth responses, such as parental underestimation, parental overestimation, and parent-youth agreement on ECV occurrence or non-occurrence. Based on these nuanced patterns of parent-youth reports of ECV, there was evidence that both parent-youth discrepancies, as well as parent-youth agreement on high levels of youth ECV predicted poor youth functioning. These findings have important implications for understanding parent-youth ECV reporting patterns and for future studies to extend the DiVIDE model.

## Aim 1: Identify Multiple Patterns of Parent-Youth Reports of Youth ECV

Researchers identified multiple patterns of parent-youth reports of youth ECV (e.g., parental underestimation, parental overestimation, parent-youth agreement), but the findings varied depending on the analytic approaches. Among studies that used analytic approaches such as mean difference scores and chi-squared tests, studies identified more frequent patterns of parent underestimation compared to parent overestimation (Howard et al., 1999; Zimmerman, 2014), as suggested by the previous literature (Goodman et al., 2010). Most of these studies identified the parent-youth response patterns descriptively, rather than statistically. The descriptive findings about frequent patterns of parental underestimation with higher youth endorsement were also found in reports of other types of violence, such as child maltreatment (Cooley & Jackson, 2020).

For studies that examined parent-youth agreement patterns (e.g., Howard et al., 1999; Skar et al., 2021), the majority of dyads fit the agreement response pattern (for exceptions see Zimmerman & Farrell, 2013; Zimmerman & Pogarsky, 2011 who examined lifetime prevalence of witnessed violence). However, these studies rarely reported whether dyads agreed on the absence, presence, or different levels of youth ECV. The few studies that did found that the majority of dyads agreed on the absence of youth ECV (e.g., Skar et al., 2021). These distinctions are crucial as parent-youth agreement on non-exposure is likely associated with the best possible outcomes, while parent-youth agreement on high levels of ECV could be associated with a greater impact on youths’ functioning, especially in higher risk populations that may have limited capacity for parental support and access to services.

## Aim 2: Evaluate the Existing DiVIDE Model by Examining Family Functioning and Youth Functioning as Correlates

The DiVIDE model (Goodman et al., 2010) attributed parent-youth discrepancies in reports of youth ECV to youth nondisclosure and negative parent-youth relationships. Additionally, parent-youth discrepancies were posited to be linked to negative youth coping and functioning. Consistent with the theory, greater parent-youth discrepancies and parental underestimation in particular were associated with poorer family functioning that is often linked to negative parent-youth relationships (e.g., lower family support, open communication, parental warmth, higher parental hostility; Zimmerman, 2014). In particular, greater parent underestimation was linked with negative family functioning (Lewis et al., 2013; Zimmerman, 2014). However, these findings should be interpreted with extreme caution as they primarily used difference scores to examine parent-youth discrepancies in youth ECV, which undermines the significance of the findings. Additionally, studies that only considered parent-youth agreement/discrepancy without further differentiation reported that disagreement was associated with more internalizing, externalizing, and PTSD symptoms, more risk taking behaviors, and lower peer support (Alers-Rojas et al., 2020; Ceballo et al., 2001; Hill & Jones, 1997; Howard et al., 1999; Zimmerman, 2014). Other studies reported that parental underestimation was specifically associated with more delinquency, internalizing, externalizing, and trauma symptoms compared to parental overestimation (Lewis et al., 2013) and parent-youth agreement groups (Zimmerman & Farrell, 2013; Zimmerman & Pogarsky, 2011).

Yet, three studies found that parent-youth agreement on the occurrence or higher frequency of ECV was associated with greater psychopathology, higher post-traumatic stress symptoms, greater impairment, and greater need for counseling services compared to parent-youth agreement on ECV non-occurrence or parent-youth discrepancies (Lewis et al., 2012; Skar et al., 2021; Turnidge-Halvorson, 2018). It is possible that when parents and youth were both aware of youth ECV, the ECV was more severe and/or led to more pronounced negative youth outcomes. Additionally, Goodman (2013) found that the association between the discrepancy pattern and youth outcome might depend on the source and developmental timing of the youth outcome information. These findings are based on studies that utilized recommended analytic methods for parent-youth discrepancies.

## Aim 3: Make Recommendations for Future Studies to Extend the DiVIDE Model

**Fig. 3:**
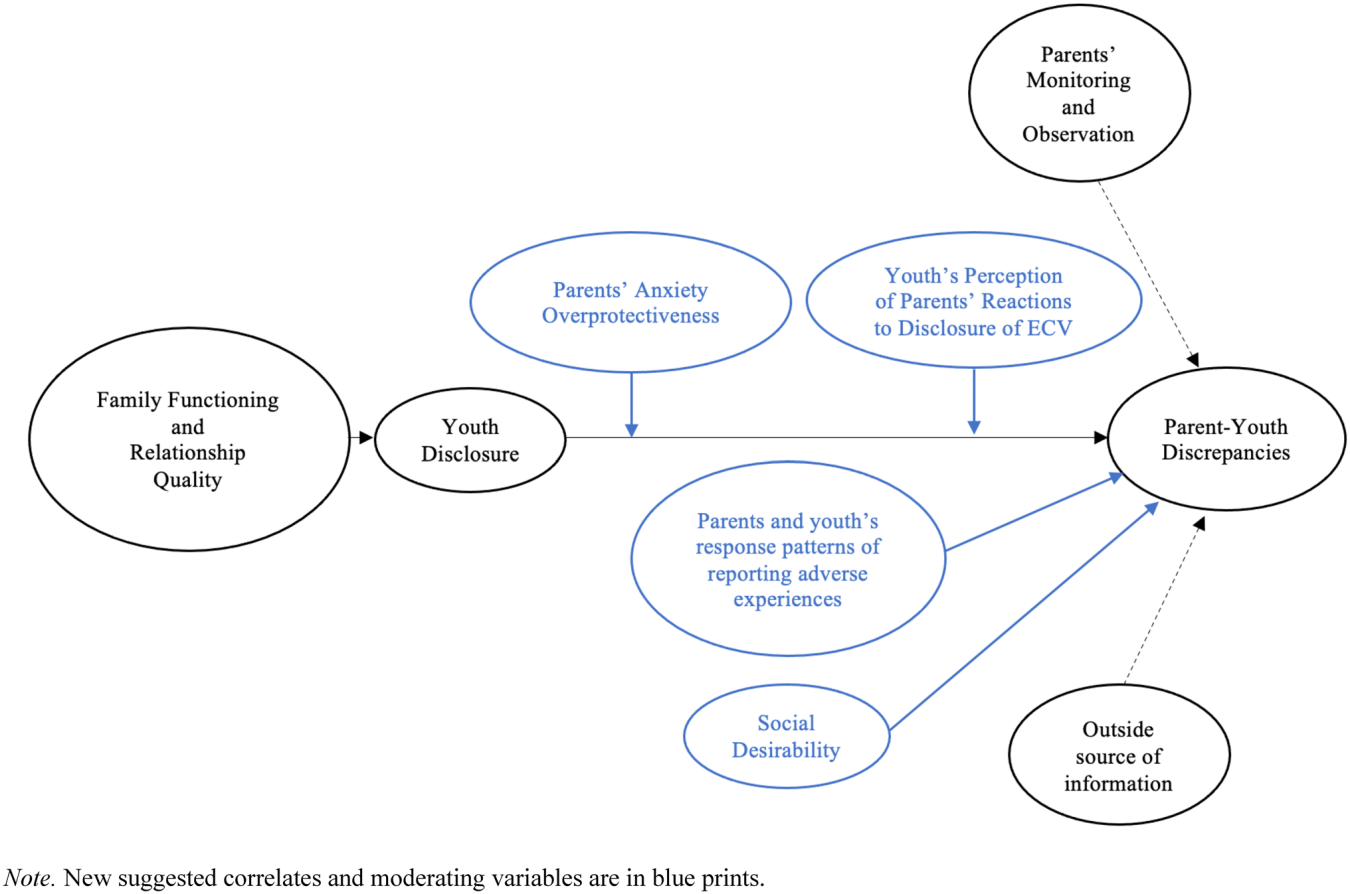
Suggestions for extending the precipitating factors in the DiVIDE model for future studies

Despite some evidence supporting the DiVIDE model’s theorization of parent-youth discrepancy reports of youth ECV, much remains to be explored. In future studies investigating youth ECV reporting discrepancies, we recommend incorporating the following additional variables as precipitating and outcome variables to test the DiVIDE model. Precipitating factors should include factors associated with youth disclosure, such as youth’s response patterns of reporting adverse experiences and social desirability. Youth exhibiting high social desirability and underreporting tendencies might be more motivated to conceal their ECV experiences. Parents’ anxiety and youth’s perception of parents’ reactions to the disclosure of ECV may also impact youth disclosure. Youth who anticipate negative reactions from parents (e.g., high anxiety, over-reaction) may choose not to disclose their ECV. Lastly, these factors should be considered within the broader cultural context, including youth age, gender identity, and their families' cultural backgrounds. Although demographic correlates of parent-youth discrepancy patterns yielded mixed findings overall, with the notable exception of youth age (i.e., older youth-parent dyads reported greater discrepancies), further research is warranted to investigate whether boys, girls, or transgender youth might be more or less likely to disclose ECV to their families or if parents are more vigilant about youth’s ECV depending on gender. Additionally, cultural factors such as cultural norms about youth disclosing adverse experiences to family members should be considered. A simplified illustration of suggestions for future studies to examine the DiVIDE model is shown in Fig. 3.

**Fig. 4:**
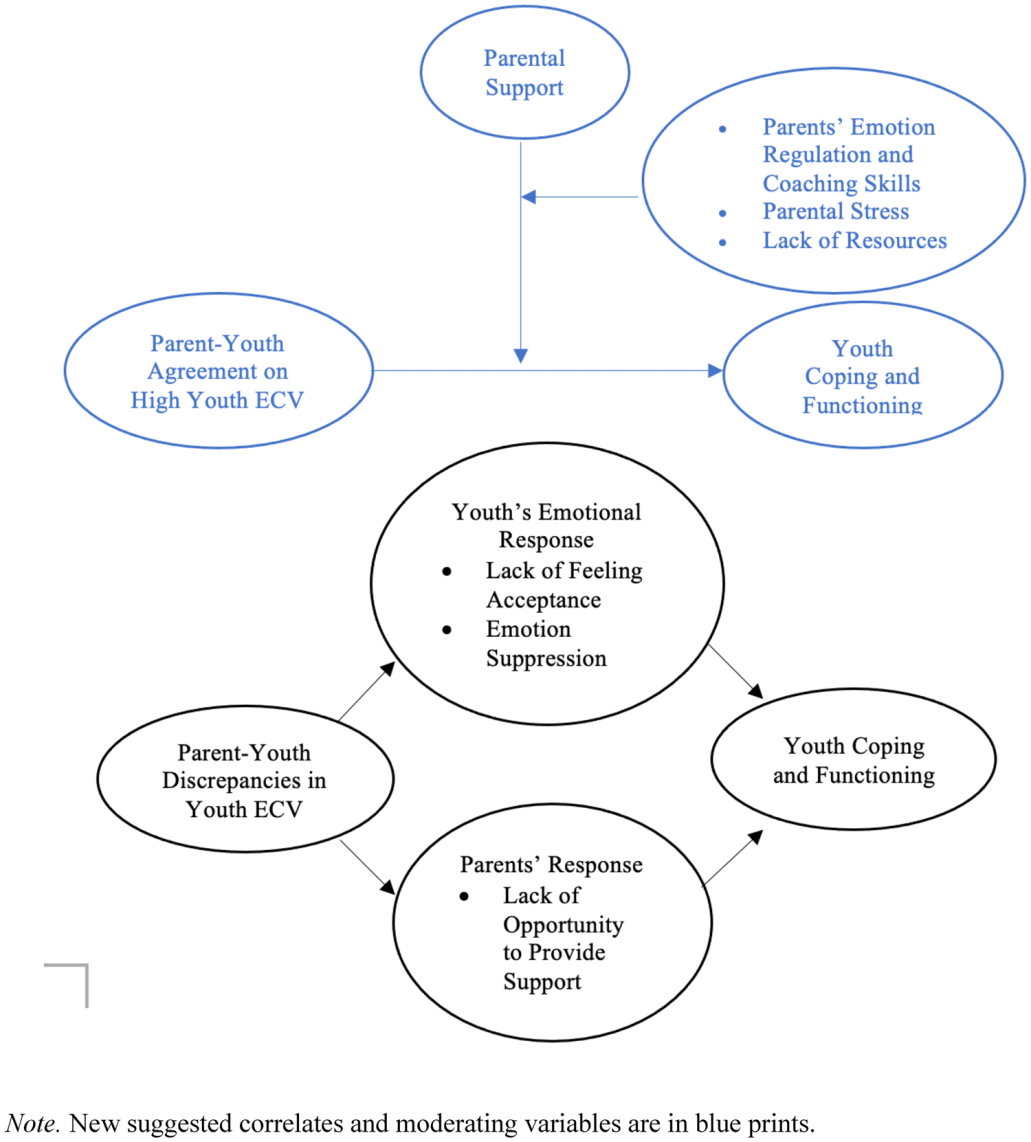
Suggestions for extending the outcome variables in the DiVIDE model for future studies

The DiVIDE model should also consider both *parent-youth agreement on high levels of youth ECV* and *parent-youth discrepancies in reports of youth ECV* as predictors of poor youth functioning based on previous findings (e.g., Zimmerman, 2014). Agreement on high levels of youth ECV might indicate severe and/or frequent exposure and could be associated with the poorest youth coping and functioning. Although the DiVIDE model suggested that parent-youth agreement on youth ECV creates opportunities for parents to support their youth, it assumed supportive parental reactions and provision of support. However, despite parent-youth agreement on the presence of youth ECV, Lewis et al. (2012) found that increased reports of needs did not necessarily translate into the utilization of counseling services. These findings suggest that potential barriers to parental support, including parent emotional reactivity, parental stress, and a lack of access to resources may influence parents’ reactions to disclosure and provision of support. In Fig. 4, we introduced parent-youth agreement on high levels of youth ECV as a separate variable that can lead to changes in youth coping and functioning. We positioned parents’ response to ECV as a moderator in the association between parent-youth agreement on high youth ECV and youth coping/functioning, recognizing that parents' response to youth ECV may lead to varying child outcomes. Factors such as parents' emotion regulation and coaching skills, parental stress, and access to resources were also considered as they may change parents’ reactions to youth ECV and the provision of support.

## Limitations of the Literature and Future Directions

Two studies using LONGSCAN datasets included the same cohorts (Lewis et al., 2012; Turnidge-Halvorson, 2018), and four studies using PHDCN datasets overlapped in cohorts and waves of studies, providing limited generalizability (Alers-Rojas et al., 2020; Goodman, 2013; Zimmerman, 2014; Zimmerman & Farrell, 2013). We suggest that future studies examine parent-youth discrepancies in youth ECV in additional samples from diverse populations. Additionally, only two studies (Goodman, 2013; Turnidge-Halvorson, 2018) used recommended data analytic methods for examining reporting discrepancies (Laird & De Los Reyes, 2013; Shanock et al., 2010). Future studies examining parent–child discrepancy reports should follow the recommendation of only using difference scores descriptively and not in correlation with other variables. Instead, these studies should use analytic approaches to identify more nuanced patterns of parent-youth discrepancies in youth ECV accurately in correlational or covariance analyses, such as person-centered analyses and polynomial regression with interaction terms. Lastly, future studies should report different dimensions of youth ECV, such as the frequency or severity of youth ECV in each group, especially if the parent-youth agreement pattern is identified. This may require the addition of follow-up questions to existing validated ECV measures.

## Limitations of the Current Systematic Review

This systematic review is not without limitations. The current review excluded studies and findings that reported parent-youth agreement in youth ECV with shared variance metrics (e.g., kappa statistics, correlation *r*), as Goodman et al. (2010) synthesized and reported most of these findings and these metrics provide limited information about parent-youth discrepancy patterns. Moreover, the current review did not consider discrepancies with other informants of youth ECV, such as teachers, clinicians, or police/court reports of youth ECV given the scope of the review and concerns about drastic underestimation of occurrence based on more distal reporters. However, discrepancies/agreements among multiple reporters (e.g., triads) should be considered in future research. It is also important to note that the preponderance of extant studies, and the DiVIDE model, assume that youth reports of ECV are accurate, attributing discrepancies to parents’ inaccurate reporting (e.g., underestimation or overestimation) or lack of knowledge. However, it is possible that youth underreport their ECV for a variety of reasons, such as social desirability, embarrassment, shame, fear of burdening or upsetting parents, or deceit. On the other hand, some youth may embellish their experiences of ECV. Lastly, this review specifically addressed discrepancies in reports of ECV and did not consider discrepancies in reports of other forms of violence that youth might encounter, such as peer victimization at school, exposure to violence in media, and cybervictimization. While we acknowledge that exposure to other forms of violence is a prevalent and serious concern, it is important to note that the decision to narrowly focus on youth’s ECV in this review was deemed necessary given the operationalization of ECV in the literature. It was also deemed appropriate, as there are likely unique differences in how parents learn of youths’ exposure to other forms of violence. For instance, parents and youth might exhibit fewer discrepancies in reporting school violence because parents may receive information about incidents of violence at school more frequently from teachers or school staff, compared to violence in the neighborhood. For cyberbullying, there could be more pronounced discrepancies between parents and youth due to the generation gap, with parents struggling to keep pace with social media dynamics. Parents may also be aware of their youth’s exposure to violence in the media that is widely circulated and available. However, future studies should also consider examining reporting discrepancies on other forms of violence.

## Conclusion

In addition to the general pattern of parental underestimation in reports of youth ECV identified in the past literature, several studies reported other patterns of parent-youth reports of youth ECV, including parental overestimation and parent-youth agreement on low or high levels of youth ECV. These patterns of parent-youth responses are important to delineate, as they were differentially associated with youth and family functioning. Some studies identified that parental underestimation was associated with lower parental warmth, support, and monitoring and more youth internalizing and externalizing problems and delinquency (Lewis et al., 2012, 2013; Zimmerman, 2014; Zimmerman & Farrell, 2013; Zimmerman & Pogarsky, 2011), as proposed in the DiVIDE model. However, other studies found that parental agreement on high levels of youth ECV was associated with more internalizing and externalizing problems and post-traumatic stress symptoms than parent-youth discrepancies (Skar et al., 2021; Turnidge-Halvorson, 2018), although findings from some studies were mixed (Goodman, 2013). Based on these findings, we recommend extending the DiVIDE model to include additional parent (e.g., hypervigilance, anxiety), youth (e.g., age, gender, social desirability), and family (e.g., cultural norms) contributors to parent-youth agreement/discrepancies in youth ECV reporting. We also recommend modifications to the model to account for additional factors that could impact parental responses to youth ECV, such as parents’ capacity to provide support and their access to services when needs are identified. Finally, we recommend that researchers use more advanced analytic strategies for investigating parent-youth reports of youth ECV (e.g., LCA, polynomial regression), as few studies used these strategies, despite their more frequent use in other fields (e.g., Becker-Haimes et al., 2017; Xu et al., 2017).

## Supplementary Information

Below is the link to the electronic supplementary material.

Supplementary file1 (DOCX 18 kb)

## Data Availability

No datasets were generated or analyzed during the current study.

## References

[CR1] Alers-Rojas, F., Jocson, R. M., Cranford, J., & Ceballo, R. (2020). Latina mothers’ awareness of their children’s exposure to community violence. *Hispanic Journal of Behavioral Sciences*, *42*(3), 324–343. 10.1177/0739986320927512

[CR2] Becker-Haimes, E. M., Jensen-Doss, A., Birmaher, B., Kendall, P. C., & Ginsburg, G. S. (2017). Parent–youth informant disagreement: Implications for youth anxiety treatment. *Clinical Child Psychology and Psychiatry,**23*(1), 42–56. 10.1177/1359104516689586 28191794 10.1177/1359104516689586 PMC5988273

[CR3] Brennan, R. T., Molnar, B. E., & Earls, F. (2007). Refining the measurement of exposure to violence (ETV) in urban youth. *Journal of Community Psychology,**35*(5), 603–618. 10.1002/jcop.20167

[CR4] Bronfenbrenner, U. (1994). Ecological models of human development. *International Encyclopedia of Education,**3*(2), 37–43.

[CR5] Buka, S. L., Stichick, T. L., Birdthistle, I., & Earls, F. J. (2001). Youth exposure to violence: Prevalence, risks, and consequences. *American Journal of Orthopsychiatry,**71*(3), 298–310. 10.1037/0002-9432.71.3.298 11495332 10.1037/0002-9432.71.3.298

[CR6] Ceballo, R., Dahl, T. A., Aretakis, M. T., & Ramirez, C. (2001). Inner-city children's exposure to community violence: How much do parents know? *Journal of Marriage and Family*, *63*(4), 927–940. 10.1111/j.1741-3737.2001.00927.x

[CR7] Chan, K. L. E. (2015). Are parents reliable in reporting child victimization? Comparison of parental and adolescent reports in a matched Chinese household sample. *Child Abuse & Neglect*. 10.1016/j.chiabu.2014.11.001 10.1016/j.chiabu.2014.11.001 25465317

[CR8] Cooley, D. T., & Jackson, Y. (2020). Informant discrepancies in child maltreatment reporting: A systematic review. *Child Maltreatment,**27*(1), 126–145. 10.1177/1077559520966387 33054358 10.1177/1077559520966387

[CR9] Cooley-Quille, M. R., Turner, S. M., & Beidel, D. C. (1995). Emotional impact of children’s exposure to community violence: A preliminary study. *Journal of the American Academy of Child & Adolescent Psychiatry,**34*(10), 1362–1368. 10.1097/00004583-199510000-00022 7592274 10.1097/00004583-199510000-00022

[CR10] De Los Reyes, A. (2011). Introduction to the special section: More than measurement error: Discovering meaning behind informant discrepancies in clinical assessments of children and adolescents. *Journal of Clinical Child & Adolescent Psychology,**40*(1), 1–9. 10.1080/15374416.2011.533405 21229439 10.1080/15374416.2011.533405

[CR11] De Los Reyes, A., Cook, C. R., Gresham, F. M., Makol, B. A., & Wang, M. (2019). Informant discrepancies in assessments of psychosocial functioning in school-based services and research: Review and directions for future research. *Journal of School Psychology*, *74*, 74–89. 10.1016/j.jsp.2019.05.005 10.1016/j.jsp.2019.05.005 31213233

[CR12] De Los Reyes, A., & Epkins, C. C. (2023). Introduction to the special issue. A dozen years of demonstrating that informant discrepancies are more than measurement error: Toward guidelines for integrating data from multi-informant assessments of youth mental health. *Journal of Clinical Child & Adolescent Psychology*, *52*(1), 1–18. 10.1080/15374416.2022.2158843 10.1080/15374416.2022.2158843 36725326

[CR13] Earls, F., & Visher, C. A. (1997). *Project on Human Development in Chicago Neighborhoods: A research update*. U.S. Dept. of Justice, Office of Justice Programs, National Institute of Justice.

[CR14] Earls, F. J., Brooks-Gunn, J., Raudenbush, S. W., & Sampson, R. J. (2007). *Project on human development in Chicago neighborhoods: Community survey, 1994–1995* (Inter-university Consortium for Political and Social Research [distributor]. 10.3886/ICPSR02766.v3

[CR15] Edwards, J. R. (2002). Alternatives to difference scores: Polynomial regression analysis and response surface methodology. In *Measuring and analyzing behavior in organizations: Advances in measurement and data analysis.* (pp. 350–400). Jossey-Bass.

[CR16] Fowler, P., Tompsett, C., Braciszewski, J., Tiura, A., & Baltes, B. (2009). Community violence: A meta-analysis on the effect of exposure and mental health outcomes of children and adolescents. *Development and Psychopathology,**21*, 227–259. 10.1017/S0954579409000145 19144232 10.1017/S0954579409000145

[CR17] Goodman, K., L. (2013). Parent-youth discrepancies in ratings of youth victimization: Associations with psychological adjustment. *American Journal of Orthopsychiatry*, *83*(1), 37–46. 10.1111/ajop.12010 10.1111/ajop.12010 23330621

[CR18] Goodman, K., L. , Reyes, A. D. L., & Bradshaw, C., P. (2010). Understanding and using informants’ reporting discrepancies of youth victimization: A conceptual model and recommendations for research. *Clinical Child and Family Psychology Review*. 10.1007/s10567-010-0076-x 10.1007/s10567-010-0076-x 20799062

[CR19] Guterman, N. B., Cameron, M., & Staller, K. (2000). Definitional and measurement issues in the study of community violence among children and youths. *Journal of Community Psychology,**28*(6), 571–587. 10.1002/1520-6629(200011)28:6%3c571::AID-JCOP3%3e3.0.CO;2-Q

[CR20] Hill, H. M., & Jones, L. P. (1997). Children's and parents' perceptions of children's exposure to violence in urban neighborhoods. *Journal of the National Medical Association*, *89*(4), 270–276. https://pubmed.ncbi.nlm.nih.gov/9145632 PMC2608202 9145632

[CR21] Hou, Y., Benner, A. D., Kim, S. Y., Chen, S., Spitz, S., Shi, Y., & Beretvas, T. (2020). Discordance in parents’ and adolescents’ reports of parenting: A meta-analysis and qualitative review. *American Psychologist,**75*(3), 329–348. 10.1037/amp0000463 31192619 10.1037/amp0000463 PMC10624508

[CR22] Howard, D. E., Cross, S. I., Li, X., & Huang, W. (1999). Parent–youth concordance regarding violence exposure: Relationship to youth psychosocial functioning. *Journal of Adolescent Health*, *25*(6), 396–406. 10.1016/S1054-139X(99)00102-0 10.1016/s1054-139x(99)00102-0 10608579

[CR23] Laird, R. D., & De Los Reyes, A. (2013). Testing informant discrepancies as predictors of early adolescent psychopathology: Why difference scores cannot tell you what you want to know and how polynomial regression may. *Journal of Abnormal Child Psychology,**41*(1), 1–14. 10.1007/s10802-012-9659-y 22773360 10.1007/s10802-012-9659-y

[CR24] Laird, R. D., & Weems, C. F. (2011). The equivalence of regression models using difference scores and models using separate scores for each informant: Implications for the study of informant discrepancies. *Psychological Assessment,**23*(2), 388–397. 10.1037/a0021926 21319905 10.1037/a0021926

[CR25] Laursen, B., & Hoff, E. (2006). Person-centered and variable-centered approaches to longitudinal data. *Merrill-Palmer Quarterly*, *52*(3), 377–389. http://www.jstor.org/stable/23096200

[CR26] Lewis, T. L., Thompson, R., Kotch, J. B., Proctor, L. J., Litrownik, A. J., English, D. J., Runyan, D. K., Wiley, T. R. A., & Dubowitz, H. (2012). Parent-youth discordance about youth-witnessed violence: Associations with trauma symptoms and service use in an at-risk sample. *Child Abuse & Neglect*. 10.1016/j.chiabu.2012.09.009 10.1016/j.chiabu.2012.09.009 PMC3762220 23153569

[CR27] Lewis, T. L., Thompson, R., Kotch, J. B., Proctor, L. J., Litrownik, A. J., English, D. J., Runyan, D. K., Wiley, T. R. A., & Dubowitz, H. (2013). Correlates of parent-youth discordance about youth-witnessed violence: A brief report. *Violence & Victims*. 10.1891/0886-6708.vv-d-12-00053 10.1891/0886-6708.vv-d-12-00053 24364128

[CR28] Magnusson, D. (2003). The person approach: Concepts, measurement models, and research strategy. *New Directions for Child and Adolescent Development,**2003*(101), 3–23. 10.1002/cd.79 10.1002/cd.79 15460974

[CR29] Moola, S., Munn, Z., Tufanaru, C., Aromataris, E., Sears, K., Sfetcu, R., Currie, M., Qureshi, R., Mattis, P., Lisy, K., & Mu, P.-F. (2020). Chapter 7: Systematic reviews of etiology and risk. In E. Aromataris & Z. Munn (Eds.), *JBI manual for evidence synthesis*. JBI. https://synthesismanual.jbi.global

[CR30] Page, M. J., McKenzie, J. E., Bossuyt, P. M., Boutron, I., Hoffmann, T. C., Mulrow, C. D., Shamseer, L., Tetzlaff, J. M., Akl, E. A., Brennan, S. E., Chou, R., Glanville, J., Grimshaw, J. M., Hróbjartsson, A., Lalu, M. M., Li, T., Loder, E. W., Mayo-Wilson, E., McDonald, S., & Moher, D. (2021). The PRISMA 2020 statement: An updated guideline for reporting systematic reviews. *British Medical Journal*, *372*, n71. 10.1136/bmj.n71 10.1136/bmj.n71 PMC8005924 33782057

[CR31] Raviv, A., Erel, O., Fox, N. A., Leavitt, L. A., Raviv, A., Dar, I., Shahinfar, A., & Greenbaum, C. W. (2001). Individual measurement of exposure to everyday violence among elementary schoolchildren across various settings. *Journal of Community Psychology,**29*(2), 117–140. 10.1002/1520-6629(200103)29:2%3c117::AID-JCOP1009%3e3.0.CO;2-2

[CR32] Runyan, D. K., Curtis, P. A., Hunter, W. M., Black, M. M., Kotch, J. B., Bangdiwala, S., Dubowitz, H., English, D., Everson, M. D., & Landsverk, J. (1998). LONGSCAN: A consortium for longitudinal studies of maltreatment and the life course of children. *Aggression and Violent Behavior,**3*(3), 275–285. 10.1016/S1359-1789(96)00027-4

[CR33] Shanock, L. R., Baran, B. E., Gentry, W. A., Pattison, S. C., & Heggestad, E. D. (2010). Polynomial regression with response surface analysis: A powerful approach for examining moderation and overcoming limitations of difference scores. *Journal of Business and Psychology,**25*(4), 543–554. 10.1007/s10869-010-9183-4

[CR34] Skar, A.-M.S., Jensen, T. K., & Harpviken, A. N. (2021). Who reports what? a comparison of child and caregivers’ reports of child trauma exposure and associations to post-traumatic stress symptoms and functional impairment in child and adolescent mental health clinics. *Research on Child and Adolescent Psychopathology,**49*(7), 919–934. 10.1007/s10802-021-00788-y 33625640 10.1007/s10802-021-00788-y PMC8154822

[CR35] Strudler, A. (2003). *Exposure to community violence by children in a residential setting* (Publication Number 3118084) [Ph.D., Fairleigh Dickinson University]. ProQuest Dissertations & Theses Global. Ann Arbor. https://login.ezproxy.lib.utah.edu/login?url=https://www.proquest.com/dissertations-theses/exposure-community-violence-children-residential/docview/305291022/se-2?accountid=14677

[CR36] Turnidge-Halvorson, N. (2018). *Informant discrepancies in youth-witnessed violence: Predictors and outcomes* (Publication Number AAI10275870) [Ph.D., The University of Utah]. APA PsycInfo®.

[CR37] Wohlin, C. (2014). Guidelines for snowballing in systematic literature studies and a replication in software engineering. In *Proceedings of the 18th international conference on evaluation and assessment in software engineering*, London, England, United Kingdom. 10.1145/2601248.2601268

[CR38] Wright, A. W., Austin, M., Booth, C., & Kliewer, W. (2017). Systematic review: Exposure to community violence and physical health outcomes in youth. *Journal of Pediatric Psychology,**42*(4), 364–378. 10.1093/jpepsy/jsw088 27794530 10.1093/jpepsy/jsw088

[CR39] Xu, Y., Boyd, R. C., Butler, L., Moore, T. M., & Benton, T. D. (2017). Associations of parent-adolescent discrepancies in family cohesion and conflict with adolescent impairment. *Journal of Child and Family Studies,**26*(12), 3360–3369. 10.1007/s10826-017-0825-2

[CR40] Zimmerman, G. M. (2014). The covariates of parent and youth reporting differences on youth secondary exposure to community violence. *Journal of Youth and Adolescence,**43*, 1576–1593. 10.1007/s10964-014-0099-6 24469322 10.1007/s10964-014-0099-6

[CR41] Zimmerman, G. M., & Farrell, A. S. (2013). Gender differences in the effects of parental underestimation of youths’ secondary exposure to community violence. *Journal of Youth and Adolescence*, *42*(10), 1512–1527. 10.1007/s10964-012-9897-x 10.1007/s10964-012-9897-x 23277295

[CR42] Zimmerman, G. M., & Pogarsky, G. (2011). The consequences of parental underestimation and overestimation of youth exposure to violence. *Journal of Marriage and Family,**73*(1), 194–208. 10.1111/j.1741-3737.2010.00798.x

