# Supplementary material for: Systematic Review of Parent-Youth Discrepancies in Exposures to Community Violence
Source: Clin Child Fam Psychol Rev. 2025 Aug 11;28(3):708–34. doi: 10.1007/s10567-025-00532-8 (PMC12634708; doi:10.1007/s10567-025-00532-8)
Supplement: Supplementary file 1 — Supplementary file1 (DOCX 18 kb) [file 10567_2025_532_MOESM1_ESM.docx]

**Supplementary Table 1**

MODIFIED JBI Critical Appraisal Checklist for analytical cross sectional studies

|  | Yes | No | Unclear | Not applicable |
| --- | --- | --- | --- | --- |
| 1. Were the criteria for inclusion in the sample clearly defined? | □ | □ | □ | □ |
| 1. Were the study subjects and the setting described in detail? | □ | □ | □ | □ |
| 1. Was the exposure (e.g., youth ECV) measured in a valid and reliable way? | □ | □ | □ | □ |
| 1. Were confounding factors identified (among studies that examined correlates of parent-youth discrepancies in youth ECV)? | □ | □ | □ | □ |
| 1. Were strategies to deal with confounding factors stated? | □ | □ | □ | □ |
| 1. Were the outcomes (e.g., youth psychopathology) measured in a valid and reliable way? | □ | □ | □ | □ |
| 1. Was appropriate statistical analysis used (e.g., analysis for examining parent-youth discrepancies in youth ECV)? | □ | □ | □ | □ |

**Supplementary Table 2**

MODIFIED JBI Critical Appraisal Checklist for cohort (Longitudinal) studies

|  | Yes | No | Unclear | Not applicable | |
| --- | --- | --- | --- | --- | --- |
| 1. Were the criteria for inclusion in the sample clearly defined? (added from JBI checklist on cross-sectional studies) | □ | □ | □ | □ |  |
| 1. Were the study subjects and the setting described in detail? (added from JBI checklist on cross-sectional studies) | □ | □ | □ | □ |  |
| 1. Was the exposure (e.g., youth ECV) measured in a valid and reliable way? | □ | □ | □ | □ |  |
| 1. Were confounding factors identified (among studies that examined correlates of parent-youth discrepancies in youth ECV)? | □ | □ | □ | □ |  |
| 1. Were strategies to deal with confounding factors stated? | □ | □ | □ | □ |  |
| 1. Were the outcomes (e.g., youth psychopathology) measured in a valid and reliable way? | □ | □ | □ | □ |  |
| 1. Were the groups/participants free of the outcome (e.g., youth psychopathology) at the start of the study (or at the moment of exposure) or did authors address the effect of outcome variable at the start of the study? | □ | □ | □ | □ |  |
| 1. Was the follow up time reported and sufficient to be long enough for outcomes to occur? | □ | □ | □ | □ |  |
| 1. Was follow up complete, and if not, were the reasons to loss to follow up described and explored? | □ | □ | □ | □ |  |
| 1. Were strategies to address incomplete follow up utilized? | □ | □ | □ | □ |  |
| 1. Was appropriate statistical analysis used (e.g., analysis for examining discrepancies in youth ECV)? | □ | □ | □ | □ |  |
